# Research progress on the effects of traditional Chinese medicine processing on the structure, content and biological activity of polysaccharides in traditional Chinese medicines

**DOI:** 10.3389/fchem.2025.1710644

**Published:** 2026-01-02

**Authors:** Jian Guan, Jiaxu Zhang, Xinzhu Tian, Jinhao Xue, Ruiying Gao, Ajiao Hou, Haixue Kuang, Liu Yang, Hai Jiang

**Affiliations:** Key Laboratory of Basic and Application Research of Beiyao (Heilongjiang University of Chinese Medicine), Ministry of Education, Harbin, China

**Keywords:** polysaccharides, processing method, content, structure, biological activity

## Abstract

Processing is the core traditional technology to regulate the efficacy of traditional Chinese medicine. Polysaccharides serve as key components in exerting biological activities such as immune regulation, antioxidant, and blood sugar reduction, its content and structural characteristics determine its biological activity, therefore, clarifying the mechanism by which processing affects the polysaccharides in traditional Chinese medicine is an important direction for explaining the processing of traditional Chinese medicine. In this paper, the main processing methods such as stir-frying, roasting and steaming are arranged and expounded in detail in terms of content, structure and activity. The results indicate that the processing affects the polysaccharide content through changing the physical properties of herbs, damaging cell structures, and triggering chemical reactions through multiple pathways. By breaking glycosidic bonds under the action of heat, acid and water, the changes of molecular weight, monosaccharide composition, functional group ratio and the spatial structure of polysaccharide were changed, thus affecting the biological activities of polysaccharide such as immunity and antioxidation. The existing research shows that the effect of processing on traditional Chinese medicine polysaccharide has the specificity of “process-medicine-component”, in the future, modern analytical techniques such as X-ray diffraction and high-resolution mass spectrometry should be combined to deeply analyze the molecular mechanism of regulating the structure-activity relationship of polysaccharides in processing, so as to provide scientific basis for the standardization and accurate optimization of processing technology of traditional Chinese medicine.

## 1 Introduction

The efficacy of traditional Chinese medicine (TCM) mainly stems from its complex chemical components. Among them, polysaccharides, as an important class of biologically active macromolecules, are one of the key material bases for the pharmacological effects of TCM (Prasher et al., 2021; Wu and Liu, 2024). Polysaccharides are widely present in both animals and plants. Common types include homopolysaccharides composed of a single monosaccharide, such as starch and cellulose, and heteropolysaccharides composed of multiple monosaccharides or their derivatives, such as pectin and glycoproteins (Tudu and Samanta, 2023; Yin et al., 2019). According to different sources, TCM polysaccharides can be classified into plant polysaccharides and animal polysaccharides. Plant polysaccharides are mainly extracted from the roots, stems, leaves and fruits of plants, such as *Lycium* chinense polysaccharides and *Aloe* polysaccharides; animal polysaccharides mainly come from the tissues, body fluids or secretions of animals, such as sea cucumber polysaccharides and chitosan (Wang H. Y. et al., 2013; Zhang et al., 2020). There are certain structural differences between the two: plant polysaccharides are mostly composed of neutral monosaccharides, while animal polysaccharides often contain amino or acidic groups (Singh et al., 2021). Despite this, both are natural polymer compounds and their biological activities can be further optimized through structural modification, such as degradation and introduction of functional groups. Compared with traditional chemical drugs, polysaccharide components have advantages such as wide sources, few side effects, and diverse pharmacological activities. They have shown broad application prospects in immune regulation, antioxidation, anti-tumor, and hypoglycemic aspects (Zheng et al., 2022). Therefore, polysaccharides have become one of the hotspots in the research and development of modern Chinese medicine.

Most TCM are derived from animals and plants. However, due to their hard texture, impurities or certain toxicity, they usually need to be processed before being applied in clinical practice (Kalita et al., 2022). Processing is an important step in the application of traditional Chinese medicines. It not only enhances efficacy and reduces toxicity, but also moderates the medicinal properties, corrects taste, and facilitates storage and formulation. Therefore, the processing techniques directly affect the content, structure and pharmacological activity of polysaccharides in TCM (Benalaya et al., 2024; Wang Z. et al., 2021).

Chinese herbal processing is a traditional processing technique guided by the theories of TCM. The *“Outline of the Development Strategy for Traditional Chinese Medicine (2016–2030)”* released in 2016 explicitly emphasized the inheritance and development of traditional pharmaceutical techniques such as processing (Liu et al., 2023). Common processing methods include purifying processing, slicing, stir-frying, steaming, boiling, etc. These processing methods can significantly alter the extraction rate, molecular weight, monosaccharide composition, functional groups and spatial conformation of polysaccharides through heat, enzymatic or chemical reactions, thereby affecting their solubility, viscosity and biological activity.

In recent years, research on polysaccharides has received increasing attention (Balducci et al., 2023), since 2000, significant progress has been made in the extraction, separation, structural identification and activity evaluation of relevant literature. However, there is still a lack of systematic reviews on the effects of different processing methods on the polysaccharides of TCM. This article aims to summarize the effects of processing methods such as stir-frying, roasting, steaming, boiling and fermentation on the content, structure, physicochemical properties and biological activities of representative polysaccharides of TCM, reveal the modification rules and mechanisms, and provide a scientific basis for the optimization of processing techniques and clinical use, as well as provide a reference for future research directions in this field (Chen et al., 2021; Chen S. et al., 2022).

## 2 Materials and methods

To comprehensively obtain the Chinese and English literature on how processing affects the physicochemical properties of polysaccharides in TCM, we conducted a systematic search in multiple Chinese and English databases, including CNKI, Wanfang, VIP, PubMed, Web of Science, etc. The search strategy was carried out by combining subject terms and free terms, using the following search formula (take Web of Science as an example):TS = ((polysaccharide* OR “plant polysaccharide” OR “animal polysaccharide”) AND (“processing” OR “traditional Chinese medicine processing” OR “paozhi” OR “herbal preparation”)). At the same time, manual retrieval and related literature tracing methods are also adopted to cover as many relevant documents as possible. The search time range is from 2000 to 2025. The search results are initially screened by browsing the titles, abstracts and keywords of the literature, and then information related to the influence of processing techniques on the physicochemical properties of polysaccharides such as solubility, molecular weight, changes in functional groups, etc. is further extracted.

## 3 The influence of processing on the polysaccharide content of TCM

This section will focus on the changes in polysaccharide content before and after processing methods such as stir-frying, roasting, and steaming. The detailed changes in content and possible reasons for the changes are presented in Table 1.

**TABLE 1:** Effect of different processing technology on polysaccharide content.

Processing method	Specific technology	Represents the changes in the content of drugs and polysaccharides before and after processing	The possible causes of content change are summarized	References
Fried method	Fried charcoal	AMR (41.74%) > AMRC (25.29%) CF(1.76%) > CFC(1.74%) TP > TPC RRR (19.8%) > RRRC (9.75%) ABR (6.05%) > ABRC (4.88%) ASR (44.57%) > ASRC (25.51%) GF (0.92%) > GFC(0.19%)	The high temperature of fried charcoal partially carbonized the medicinal materials, and the polysaccharide structure was severely destroyed, and the content of polysaccharide decreased due to the carbonization of polysaccharide and other sugar components	Chen et al. (1997) Kong et al. (2024) Xi and Li, (2000) Yang and Li (2010) Lin et al. (2008) Jin et al. (2000) Sheng et al. (2006)
	Bran frying	AMR (41.74) > BFAMR (26.92%) AR (23.17%) > BFAR(22.48) CR (39.69%) > BFCR(25.38%) DR (72.63%) < BFDR (76.44%)	Maillard reaction occurred in the high temperature process of bran frying, which degraded polysaccharide and led to the decrease of polysaccharide content. However, the texture of DR becomes looser after bran frying, which may also increase the dissolution of polysaccharide	Chen et al. (1997) Luo et al. (2009) Cai et al. (2008)
	Rice frying	CR (38.59) > RFCR(26.23)	Maillard reaction occurs in the high temperature process of rice frying, which leads to the degradation or transformation of polysaccharide and the decrease of polysaccharide content	Chen et al. (2019)
	Soil frying	DR (13.4%) > SLFDR (12.6%) ASR (44.57%) > SLFASR (37.38%) AMR (41.74%) > SLFAMR (29.81%)	At soil frying time, the heat transfer temperature of the soil is high, and the high temperature degrades or carbonizes the polysaccharide, which leads to the decrease of polysaccharide content	Jin et al. (2000)
	Sand frying	GGEC (0.0149%) < SFGGEC (0.0207%) CBR(28.56%) < SFCBR(36.06%)	After frying, the texture of medicinal materials is crisp, and the tissue porosity is enhanced, which is beneficial to the dissolution of effective components or the transformation of some components into polysaccharides	Jin et al. (2000) Chen et al. (2010)
	Stir-fried	AMR > STFAMR ASR > STFASR OR > STFOR PRA > STFPRA AR (4.52%) > STFAR(4.03%) AHR < STFAHR BR < STFBR CS(6.71%) < STFCS(8.27%) EF (2.043%) < STFEF (2.432%) UL (12.147 mg/g) < STFUL (37.780 mg/g)	The high temperature of frying degrades the polysaccharide or carbonizes some of the polysaccharide or converts it into reducing sugar, which reduces the content of polysaccharide. Appropriate frying temperature will promote the dissolution of polysaccharide or the break of glycopeptide bond, which may increase the content of polysaccharide. The destruction of cell wall during frying may also promote the dissolution of polysaccharide	Pan et al. (2016) Jin et al. (2000) Xue et al. (2025b) Wang et al. (2013b) Liu et al. (2016) Bai et al. (2021) Li et al. (2016) Xu et al. (2012) Yang and Hu, 2005 Xie et al. (2008)
Roasting method	Honey processing	CR (28.59%) < HPCR(42.58%) LB (1.2336%) < HPLB (2.2784%) POR (36.083%) < HPPOR (50.880%) POR (21.18%) < HPPOR (27.96%) EBF(9.16%) < HPEBF(9.51%) AR (9.95%) > HPAR(8.26%) AR (4.96%) > HPAR(4.32%) CR > HPCR	Honey processing does not rule out the influence of honey as an auxiliary material on the content of this component. When the temperature is low, the content of polysaccharide in Chinese herbal medicines can be increased. When the temperature is high, the high temperature will transform polysaccharide into oligosaccharide or some components will be denatured due to direct heating, which will reduce the content of polysaccharide	Chen et al. (2019) Zhang et al. (2006) Wang et al. (2012b) Wang et al. (2012a) Zhou et al. (2014) Guo et al. (2022) Liu et al. (2016) Tang et al. (2022)
	Wine processing	ASR (5.59%) < WASR (6.20%) ASR (5.90%) < WASR (8.53%) CBR(28.56%) < WCBR(36.06%) CF(51.41%) < WCF(53.10%) CF(3.59%) < WCF(4.27%) MOR (1.98%) < WMOR (2.64%) EF (2.043%) < WEF (2.124%) PR (65.05 mg/g) > WPR(30.5 mg/g)	Processing with wine may break a lot of cell walls, increase the degree of dissolution of polysaccharide, increase the solubility, and increase the content of polysaccharide. High temperature can also transform polysaccharide into oligosaccharide, which reduces the content of polysaccharide	Jin et al., 2000 Liu and Xia (2006) Zhao et al. (2015) Liang et al. (2009) Ding and Cai (2006) Chen et al. (2012) Yang and Hu, 2005 Guo et al. (2022)
​	Salt processing	ABR (6.05%) < SPABR (8.20%) SR (64.591 mg/g) < SPSR(64.951 mg/g) CBR(28.56%) < SPCBR(49.60%) AHR (1.53%) < SPAHR (1.98%) AHR < SPAHR MOR (1.98%) < SPMOR (2.18%) MOR (2.163%) < SPMOR (2.312%) EF (2.043%) < SPEF (2.101%) LLF (7.82%) > SPLLF (7.04%)	The addition of salt can promote the dissolution of polysaccharide to some extent, or the wall-breaking of medicinal materials can promote the dissolution of polysaccharide and increase the content of processed polysaccharide. However, the water-soluble polysaccharide of LLF was lost during the processing, and the polysaccharide content decreased	Lin et al. (2008) Wei et al. (2021) Zhao et al. (2015) Xu and Wang, 1999a Bai et al. (2021) Shi et al. (2018) Chen et al. (2012) Yang and Hu, 2005 Xu et al. (1999b)
	Vinegar processing	BR (20.47%) > VBR(19.40%) SCF(6.18%) > VSCF(5.59%)	The acidic environment of vinegar promotes the degradation of polysaccharide, which may reduce the content of polysaccharide. Different polysaccharides have different tolerance, and the content of polysaccharide decreases in different ways	Li et al. (2016) Ren et al. (2012) Xu et al., 1999b Xi and Li (2000)
	Other processing	CBF processed by RRER: CBF(2.13%) < CBF PB RRER (3.55%) EDF processed by GRER:EDF (5.01%) < EDF PB GRER (6.19%) MOR processed by GRER:MOR (1.98%) < MOR PB GRER (2.68%)	Processing may have a certain impact on the structure of TCM, which makes the polysaccharide easier to precipitate and the polysaccharide content increases. In the process of roasting licorice, it involves operations such as soaking, moistening and frying licorice juice. The licorice polysaccharide in licorice juice is adsorbed by EDF and MOR, which leads to the increase of polysaccharide content after processing	Wei et al. (2020)
Steam method	Steaming	PMR < SPMR SPR(64.89%) < SSPR(65.9%) oven-drying PAR(14.93%) < oven-drying SPAR(20.14%) ASB < SASB	High temperature processing breaks the sugar chains of monosaccharide, disaccharide, and polysaccharide, increasing water solubility, reducing viscosity, and improving the dissolution rate of polysaccharide, ultimately enhancing its biological activity	Song et al. (2017) Ren et al. (2025) Teng et al. (2022) Yu and Zhang (2006) Jia et al. (2024) Qiu et al. (2006) Fang et al. (2012) Li et al. (2022)
	Nine steaming and nine processing	White ginseng (2.4 mg/mL) < red ginseng (4.0 mg/mL) white ginseng (2.4 mg/mL) < black ginseng (4.5 mg/mL) PR (4.254%) < NSPR(10.635%) SPF(2.153%) < NSSPF(4.172%) PMR (21.22%) < NSPMR (22.9%)	The content of polysaccharide decreased gradually in the process of nine steaming and nine processing, which was related to the hydrolysis of polysaccharide into monosaccharide or oligosaccharide in the processing process, which was beneficial to the frying of effective components	Dong et al. (2025) Yu et al. (2022) Ren et al. (2020) Li et al. (2024) Lin et al. (2021) Qin et al. (2020) Zheng et al. (2020) Chen et al. (2018) Ren et al. (2025) Guan et al. (2024) Luo et al. (2025b) Jin et al. (2015) Huang et al. (2023) Yang et al. (2022b) Hu et al. (1999) Niu et al. (2024) Cheng et al. (2019)
Fermentation method	Fermentation	MYS < FMYS MYS > FMYS	In the early stage of fermentation, microbial reproduction produced a large number of enzymes, which consumed macromolecular sugars in MYS to produce soluble sugars, and the content of polysaccharides increased. In the later stage of fermentation, the decrease in the growth rate of total polysaccharides may be related to the massive growth and reproduction of microorganisms, but it consumed the total polysaccharides produced	Wang et al. (2025)

### 3.1 Fried method

Stir-frying, as an important method in traditional Chinese medicine processing, can achieve the purposes of drying, purifying, moderating the properties of the medicine, reducing toxicity or altering the physical and chemical properties through heating treatment (Lin, 1997). The influence on the content of polysaccharide components is complex and diverse, specifically depending on the stir-frying method and the type of medicinal materials. This section elaborates on the impact of various common stir-frying techniques, such as charcoal frying, bran frying, rice frying, soil frying, sand frying, and stir-frying, on the polysaccharide content of TCM (Xiaolong et al., 2023).

Most stir-frying methods cause the polysaccharides to undergo degradation, carbonization or Maillard reactions due to high temperatures, resulting in a decrease in their content (Ke and Li, 2023). For instance, during the process of roasting the charcoal, the polysaccharide content of medicinal materials such as *Atractylodis Macrocephalae Rhizoma* (AMR), *Corni Fructus* (CF), *Typhae Pollen* (TP), *Rhei Radix et Rhizoma* (RRR), *Achyranthis Bidentatae Radix* (ABR), *Angelicae Sinensis Radix* (ASR) and *Gardeniae Fructus* (GF) all decreased. After stir-frying with bran AMR (Chen et al., 2010), *Astragali Radix* (AR)、*Codonopsis Radix* (CR) (Luo et al., 2009), stir-frying with rice the CR (Chen et al., 2019), soil frying AMR (Pan et al., 2016), ASR (Jin et al., 2000) and stir-fried *Ophiopogonis Radix* (OR) (Xue Y. J. et al., 2025), *Paeoniae Radix Alba*(PRA) (Wang Y. et al., 2013), AR), their content of polysaccharides are also decreased to different extents. However, under certain stir-frying conditions, the polysaccharide content of some medicinal materials does not decrease but increases instead. For instance, bran frying *Dioscoreae Rhizoma* (DR) (Cai et al., 2008), sand frying *Galli Gigerii Endothelium Corneum* (GGEC) (Wang N. et al., 2021) and *Cibotii Rhizoma* (CBR) (Zhao et al., 2015) and stir-fried *Anemarrhenae Rhizoma* (AHR) (Bai et al., 2021), *Bupleuri Radix* (BR) (Li et al., 2016), *Cuscutae Semen* (CS) (Xu et al., 2012), *Epimedii Folium* (EF) (Yang and Hu, 2005) and *Urticadioica* L (UL) (Xie et al., 2008), their polysaccharide contents increased. Table 1 systematically summarizes the specific content changes and the reasons for such changes of the polysaccharides from different processed TCMs.

In conclusion, the impact of stir-frying on the polysaccharide content of TCM is dual: high temperatures usually lead to degradation or carbonization, resulting in a decrease in content; while at appropriate temperatures, it may increase the polysaccharide solubility and content by altering the texture of the medicinal materials, breaking down the cell walls or promoting component transformation. This finding has significant reference value for guiding the optimization of processing techniques and clinical medication.

### 3.2 Roasting method

As one of the fundamental techniques in TCM processing, the roasting method involves mixing prepared or sliced medicinal materials with specific liquid excipients-such as honey, wine, salt, and vinegar-and stir-frying them to facilitate the penetration of these excipients into the herbal matrix. This process is designed to enhance therapeutic efficacy, moderate pharmacological properties, reduce toxicity, and improve storability (Zhang et al., 2025a). The effects of different roasting methods on the polysaccharide content of TCM vary, mainly depending on the type of drug, the structure of polysaccharides, the nature of excipients and the parameters of processing technology. Overall, it can be summarized into two categories: “increased content” and “decreased content”, the possible reasons involve multiple factors such as excipients promoting dissolution, high-temperature degradation, Maillard reaction, cell wall disruption, and component adsorption (Bai et al., 2024). The following is a systematic summary of the effects of various roasting methods on polysaccharide content (Zhang et al., 2025b).

After being honey-processing, the polysaccharide content of CR (Chen et al., 2019), *Lilii Bulbus* (LB) (Zhang et al., 2006), *Polygonati Odorati Rhizoma* (POR) (Wang et al., 2012a; Wang et al., 2012b) and *Eriobotryae Folium* (EBF) (Zhou et al., 2014) increases; however, the polysaccharide content in some samples of some AR (Cai et al., 2016; Liu et al., 2016) and some CR (Chen et al., 2019) decreased after being honey-processing. Wine-basting can increase the polysaccharide content in ASR (Jin et al., 2000; Liu and Xia, 2006), CBR (Zhao et al., 2015), CF (Ding and Cai, 2006; Liang et al., 2009), *Morindae Officinalis Radix* (MOR) (Chen et al., 2012) and EF (Yang and Hu, 2005), however, the polysaccharide content of *Polygonati Rhizoma* (PR) (Guo et al., 2022) after aging treatment significantly decreases. After salt roasting, the polysaccharide contents of ABR (Lin et al., 2008), *Scutellariae Radix* (SR) (Wei et al., 2021), CBR (Zhao et al., 2015), AHR (Xu and Wang, 1999a) and EF (Yang and Hu, 2005) generally increased. However, *Ligustri Lucidi Fructus* (LLF) (Xu et al., 1999b), which contains a large amount of water-soluble polysaccharides in itself, was prone to lose its content during the salt roasting and thus its content decreased. The vinegar processing usually leads to a decrease in the content of polysaccharides, such as BR (Li et al., 2016), *Schisandrae Chinensis Fructus* (SCF) (Ren et al., 2012), LLF (Xu et al., 1999b) and TP (Xi and Li, 2000). The possible reasons may be that the acidic environment of vinegar promotes the degradation of polysaccharides, the liquid excipients inhibit the dissolution, or the heating causes the decomposition of heat-sensitive polysaccharides.

Other roasting methods [e.g., *Chebulae Fructus* (CBF) processed by *Rubiae Radix et Rhizoma* (RRER) (Wei et al., 2020), *Euodiae Fructus* (EDF) (Li et al., 2012) and MOR (Chen et al., 2012)] processed by *Glycyrrhizae Radix et Rhizoma* (GRER) all show an increase in polysaccharide content, the mechanisms include altering the structure of the medicinal materials to promote the release of polysaccharides, or the polysaccharides contained in the adjuvant (e.g., Glycyrrhiza polysaccharide) being adsorbed onto the drugs.

In conclusion, the effect of roasting on the polysaccharide content of TCM shows significant method specificity and material dependence. The mechanism of change is complex and diverse. In the actual optimization of processing techniques and quality control, a comprehensive evaluation should be conducted in combination with specific drugs and processing parameters.

### 3.3 Steaming method

Steaming is a key method in TCM processing that modifies the properties of medicinal materials through the application of moist heat generated by high-temperature steam, its primary objectives include enhancing therapeutic efficacy, reducing toxicity, moderating the pharmacological nature of the herbs, and facilitating storage (Zhou et al., 2024). The influence of this method on the content of polysaccharides in TCM is jointly regulated by factors such as the type of medicinal materials, the number of steaming times, temperature, time, and whether or not excipients are added. Overall, it shows different trends such as an increase, a decrease, or an initial increase followed by a decrease in content.

During the steaming process, *Polygoni Multiflori Radix* (PMR) (Jia et al., 2024; Qiu et al., 2006), *Scrophulariae Radix* (SPR) (Fang et al., 2012), *Pseudostellariae Radix* (PAR) (Sheng et al., 2009) and *Allii Sativi Bulbus* (ASB) (Li et al., 2022) generally increases, however, the polysaccharide content of PR (Ren et al., 2025; Song et al., 2017; Teng et al., 2022; Yu and Zhang, 2006) decreased after steaming. The nine steaming and nine processing, as a repeated steaming and drying intensification method (Liu J. et al., 2022), has a more complex impact on the polysaccharide content (Bian et al., 2022; Chang et al., 2023). For example, the polysaccharide content of PMR (Cheng et al., 2019), *Sophorae Fructus* (SPF) (Niu et al., 2024) and RRR (Hu et al., 1999) generally increases after being processed by this method, and the content often shows a trend of first rising and then falling during multiple steaming and drying, the polysaccharide content of the samples processed by steaming and sun-drying six times each is often higher. On the other hand, the polysaccharide content of PR decreases after nine steaming and nine processing (Chen et al., 2018; Dong et al., 2025; Guan et al., 2024; Li et al., 2024; Lin et al., 2021; Luo X. et al., 2025; Qin et al., 2020; Ren et al., 2020; Yu et al., 2022; Zhang Q. et al., 2024; Zheng et al., 2020). Further research on the processing of *Ginseng Radix et Rhizoma* (GRR) has revealed that the content of acidic polysaccharides in white ginseng increases with the number of steaming sessions when it is transformed into red ginseng and even black ginseng, the content of acidic polysaccharide increased with the increase of steaming times (Jin et al., 2015), however, the significant increase in reducing sugar content also indicates the decomposition of polysaccharides (Huang et al., 2023; Yang Z. et al., 2022).

In conclusion, the effect of steaming on polysaccharide content in traditional Chinese medicinal materials is highly dependent on the specific processing conditions and varies according to the type of herb. Moderate steaming can promote the accumulation of polysaccharides by destroying cell structures and inhibiting enzyme activity, however, prolonged or repeated steaming may lead to the degradation of polysaccharides due to hydrolysis and the Maillard reaction. In practical applications, it is necessary to optimize the steaming parameters to maximize the retention or enhancement of polysaccharide activity (Zhang Z. J. et al., 2024).

### 3.4 The influence of other processing methods on the polysaccharide content

Fermentation technology significantly affects the content of polysaccharides through microbial metabolism (Yu et al., 2021). For instance, the polysaccharide content of *Myristicae Semen* (MYS) increases with the extension of fermentation time, but it decreases in the later stage as the fermentation process continues (Wang et al., 2025), When comparing the MOR processed by yeast fermentation, the one processed by *Bacillus* DU-106 fermentation and the one with the core removed, it was found that the polysaccharide content increased from 2.91% to 4.03% after yeast fermentation, and from 2.91% to 3.79% after *Bacillus* DU-106 fermentation (Tan et al., 2018). It can be seen that the polysaccharide content increased after fermentation. It is speculated that this might be related to the fact that the reproduction of microorganisms after fermentation consumed the macromolecular sugars in MOR and produced soluble sugars. In addition to the above processing methods, there are also microwave processing and some local processing techniques in the processing methods. When comparing the content of *Pinelliae Rhizoma* and *Pinelliae Rhizoma Praeparatum*, it was found that the polysaccharide content of processed *Pinelliae Rhizoma* increased from 4.63% to 11.60%, this might be one of the reasons why processing *Pinelliae Rhizoma* reduces toxicity and enhances efficacy (Lai et al., 2007). The content of polysaccharides in *Citri Sarcodactylis Fructus* (CSF) is 46.72 mg/g, after microwave treatment for 90 s, the content is 47.31 mg/g; for 120 s, it is 39.90 mg/g; and for 180 s, it is 24.81 mg/g. As the microwave heating time increased, partial carbonization occurred in the medicinal materials, and the content of polysaccharides decreased (Li and Yao, 2012). The DR is processed by the Jianchang group method, which was similar to bran frying, but the auxiliary materials were changed from bran to honey bran. Compared with the unprocessed polysaccharide, the content of polysaccharide processed by Jianchang group increased from 2.82% to 4.76%. The increase in content was mainly due to the uniform heating of DR during processing, which helped the dissolution of polysaccharide and increased the content (Zheng et al., 2018).

## 4 The influence of processing on the structure of polysaccharides in TCM

The structure of polysaccharides is usually described hierarchically from “primary structure” to “higher structures” (secondary, tertiary, and quaternary). The structural characteristics of polysaccharides in TCM also follow this pattern, with the core aspects being “monosaccharide composition”, “linkage mode”, “molecular weight”, and “spatial conformation”, etc., (Xie et al., 2025). Its structure is complex and diverse, and it is the core basis for demonstrating biological activity. Meanwhile, TCM processing, through physical or chemical effects, may directly influence the structure of polysaccharides (Chen X. et al., 2022). The relevant information of representative TCM is shown in Table 2.

**TABLE 2:** Effects of different processing methods on the structure of polysaccharides from TCM.

Represents TCM	Changes of molecular weight and monosaccharide composition before and after processing	Chemical constitution	References
MMF	The molecular weight before stir-frying was 26.437 kDa; The contents of Fuc, Rha, Gal, Man, Gal UA and Man UA are high After stir-frying, the molecular weight is 15.565 kDa; The contents of Ara, Glc and Xyl are higher	The unprocessed MMF is mainly composed of β-glycosidic bonds. After frying, the content of β-glycosidic bonds is higher, and the β-glycosidic bonds are broken due to instability and new glycosidic bonds (1,4,6-linked-Glcp)are generated	Liu et al. (2024b)
	The main monosaccharides with molecular weight of 9534Da before frying are Ara, Gal, Glc, Xyl, Man, Gal UA and Glc UA. After frying, the molecular weight was 8652Da, and two monosaccharides (Glu UA) and (Man UA) were added after frying	The hydroxyl group of Gal is oxidized to generate carboxyl group of GalUA, and the hydroxyl group of Glc is oxidized to generate carboxyl group of GlcUA.	Liu et al. (2022b)
AR	The molecular weight before honey baking was 3.64 × 10^6^Da, and the main monosaccharides were Man, Gal UA, Glc, Fuc and Fru. After honey processing, the molecular weight was 2.34 × 10^6^Da, and the contents of Fru, Man and Glc increased, but the content of Gal UA decreased. The molar ratio of Man: Glc: Xyl: Ara: Glc UA: Rha = 0.27:12.83:1.63:0.71:1.04:0.56 before honey processing, and Glc: Xyl: Ara: GlcUA = 0.06:28.34:0.58:0.24:0.33:0.21 after honey processing	NA	Zhao et al. (2023) Liao et al. (2018)
SCF	The molecular weight range before vinegar processing is 1300–3100Da, and the monosaccharide composition is Rha, Glc, Gal and D-Xyl. After vinegar processing, the molecular weight range is reduced to 800–2400Da, and the monosaccharide composition is Rha and Glc	NA	Guo et al. (2024)
PR	The molecular weight before steaming is 1200kDa, and after nine steaming and nine processing, it is 970 kDa. The monosaccharide with molecular weight of 4.01 × 103Da before steaming consists of Glc and Xyl The monosaccharides with molecular weight of 1.42 × 104Da after six steaming are composed of Glc, Man and Xyl The monosaccharide composition before steaming was Man, GalUA, Glc, Gal and Ara, but after steaming, the content of Glc increased, while the content of Man, GalUA, Gal and Ara decreased Man, Glc, Gal and Ara before steaming. After nine steaming and nine processing, the content of Man decreased, the content of Glc first increased and then decreased, and the content of Gal and Ara increased. Rha, Man, Glc, Gal, Xyl and Ara before steaming, and the contents of Rha and Gal increased after nine-steaming and nine- processing; The contents of Glc and Man decreased There was a glycosidic bond β-1,4-manp before steaming, and a new glycosidic bond β-1,4-Galp appeared after steaming, and β-1,4-manp increased	PR has β-fructan structure before steaming, but after steaming, β-fructan structure is destroyed, and large aggregates are formed through intermolecular hydrogen bonds, especially the interaction between carboxyl groups of uronic acid or strongly negative oxygen atoms on carboxyl groups and hydroxyl hydrogen on other sugar chains	Zhang et al. (2024a) Wu et al. (2022b) Li et al. (2021c) Ma et al. (2024) Wu et al. (2021) Yao et al. (2024) Chen et al. (2022c)
Chlamys nobilis	NA	β-glycosidic bond Transition from β type to α type after processing	Yang et al. (2025)
CH	Before steaming, Glc: Ara: Man = 89.82:3.47:3.98, and after steaming, Glc: Ara: Man = 79.3:10.43:5.59, and the Glc content decreased obviously	Polysaccharide has triple helix structure. The triple helix structure disappears after steaming	Hou et al. (2023)
Polygonatum cyrtonema	Before steaming, Gal and Man were the main components, followed by Glc, Rib, Fuc and GlcUA were higher after six-steaming and six-processing, and Gal and Man were lower after nine-steaming and nine- processing	The triple helix structure of polysaccharide disappeared after steaming	Fan et al. (2020)
GGEC	Rha, Fuc, Man, Glc and Gal were the main components before frying, and the monosaccharide content increased after frying	NA	Wang et al. (2021a)

### 4.1 The influence of processing on the molecular weight of polysaccharides in TCM

During the processing of TCM, multiple factors such as high temperature, liquid excipients and repeated heat treatment often lead to a reduction in the molecular weight of polysaccharides (Wang et al., 2023d), the main mechanisms involved include glycosidic bond cleavage, acid-catalyzed hydrolysis and Maillard reaction. During the frying process, high temperatures can cause the breakage of polysaccharide chains and structural degradation. For instance, the weight-average molecular weight (Mw) of Massa Medicata Fermentata (MMF) decreased from 26.437 kDa to 15.565 kDa (Liu S. et al., 2024); the Mw of Massa grass decreased from 9,534 Da to 8,652 Da after being stir-fried (Liu S. et al., 2022). The high temperature and Maillard reaction during the honey processing of AR cause the degradation of polysaccharides, resulting in the molecular weight of honey processed AR polysaccharide (ARP) being greater than AR (Zhao et al., 2023). The acidic environment during vinegar processing can trigger acid-catalyzed hydrolysis of glycosidic bonds, leading to the degradation of polysaccharides into smaller molecular fragments, for instance, the molecular weight of SCF polysaccharide decreases after vinegar processing (Guo et al., 2024); the molecular weight distribution of CF vinegar processed products reduces from 1,300–3,100 Da to 800–2,400 Da. The impact of steaming on the molecular weight of polysaccharides is rather complex and often shows dynamic changes with the duration and frequency of steaming. In the processing of GRR as the intensity of steaming increases, the molecular weight of its polysaccharides initially rises and then drops, but still remains lower than that of the raw product (Jiao et al., 2014). After nine rounds of steaming and processing, the maximum molecular weight of polysaccharides in PR odoratum decreased from 1,200 kDa to approximately 970 kDa, and the minimum molecular weight dropped from 3,878 kDa to 858 kDa, moreover, the more times it is steamed, the more significant the decrease in molecular weight becomes, which might be related to the dissociation of sugar chains caused by high temperature. In some cases, specific processing conditions may cause a temporary or local increase in the molecular weight of polysaccharides. For instance, after steaming for 2 and 4 h, the molecular weight of PRP increased, and even high-molecular-weight components of (1.2–4.4) × 10^6^ Da were detected in the sample steamed for 6 h, the average molecular weight of the product steamed in wine for 6 h (1.42 × 10^4^ Da) was also higher than that of the raw material (4.01 × 10^3^ Da) (Li et al., 2021c; Wu W. J. et al., 2022). It is speculated that this might be related to the formation of aggregates through hydrogen bonds and electrostatic interactions of acidic polysaccharides in solution, however, such phenomena are not common, and the overall trend is still a decrease in molecular weight.

Overall, the effect of processing on the molecular weight of polysaccharides in TCM is mainly a reduction. The general mechanism can be summarized as follows: under the influence of heat, acid, and moisture, chemical changes such as hydrolysis of glycosidic bonds, cleavage of sugar chains, and Maillard reactions occur, leading to the degradation of polysaccharides into smaller molecular weight fragments. This process is closely related to the processing methods, temperature, and time control, and can be regarded as a universal rule for understanding the changes in the physicochemical properties of polysaccharides caused by processing.

### 4.2 The influence of processing on the monosaccharide composition of polysaccharides in TCM

Processing is an important step in the preparation of TCM, which has a significant impact on the monosaccharide composition of polysaccharides. The monosaccharide composition of polysaccharides usually includes neutral monosaccharides such as glucose (Glc), galactose (Gal), mannose (Man), Ara, rhamnose (Rha), xylose (Xyl), etc., acidic monosaccharides, such as glucuronic acid (Glc UA) and galacturonic acid, and amino sugars, such as glucosamine. Because monosaccharides themselves have stable chemical properties, processing generally does not lead to significant increases or decreases in the types of monosaccharides, but it can significantly alter the relative proportions of each monosaccharide by breaking glycosidic bonds or triggering degradation and transformation reactions.

The high temperature during the stir-frying process can trigger oxidation and structural transformation, leading to changes in the proportion of monosaccharides or even the generation of new monosaccharides. For instance, in the fried MMF, the proportion of Ara, Xyl and Glc increase, while the contents of Fuc, Rha, Gal and uronic acid decrease, this might be related to the high-temperature oxidation effect (Liu S. et al., 2024). The proportion of uronic acid in the fried product of MMF increased, suggesting that some hydroxyl groups were oxidized to carboxyl groups during the stir-frying process (Liu S. et al., 2022). After stir-frying, the content of each monosaccharide in GGEC generally increased, which might be related to the promotion of dissolution or component transformation due to the loose texture (Wang N. et al., 2021). The roasting method affects the monosaccharide composition through the combined action of excipients and high temperature. The content of fructose and Glc in honey-processed AR significantly increases, while the proportion of galacturonic acid decreases, this is mainly due to the introduction of fructose and Glc by honey and the hydrolysis and Maillard reaction of sucrose during the processing. Honey processing can also change the proportion of monosaccharides. Research shows that the proportion of Glc significantly increases after honey processing (Zhao et al., 2023). When SCF is stir-fried with vinegar, glycosidic bond hydrolysis occurs in an acidic environment, leading to changes in the content of Rha and Gal and other monosaccharides, this indicates that acidic adjuvants can promote the degradation or transformation of specific monosaccharides (Guo et al., 2024).

Steaming promotes the degradation or transformation of polysaccharides through the effect of moist heat, thereby altering the composition of monosaccharides. In steamed PR, the proportion of glucose first decreases and then increases, while the proportions of Xyl and Gal increase, reflecting the depolymerization of polysaccharides and the conversion of reducing sugars (Li et al., 2021c; Wu W. J. et al., 2022). After steaming, the Glc content of *Cistanches Herba* (CH) decreased, but the types of monosaccharides remained unchanged. In PR after nine steaming and nine processing, the proportion of glucose increased, while the proportions of Man and Gal UA (Galactouronic acid) decreased, which was closely related to the Maillard reaction and the cleavage of glycosidic bonds (Ma et al., 2024; Wu et al., 2021). In addition, an increase in the number of steaming times usually led to the decomposition of oligosaccharides into monosaccharides, such as an increase in fructose content, and the proportion of monosaccharides tended to stabilize.

Based on the above three types of processing methods, the common rules can be summarized as follows: The stir-frying method mainly promotes oxidation reactions and structural destruction through high temperatures, changes the proportion of uronic acid and the relative content of neutral monosaccharides, and overall manifests as the adjustment of the molar ratio of monosaccharides; the roasting method introduces exogenous monosaccharides through auxiliary materials such as honey and vinegar or provides an acidic environment to promote hydrolysis, degradation and Maillard reaction, thereby significantly altering the proportion of monosaccharides in polysaccharides; the steaming method, by virtue of moist heat conditions, triggers sugar chain breakage, Maillard reaction and oligosaccharide degradation, leading to a significant change in the proportion of monosaccharides, and the greater the steaming intensity, the more obvious the change.

Overall, the influence of processing on the monosaccharide composition of polysaccharides is mainly characterized by changes in proportion, and the increase or decrease in types is rare. Specifically, stir-frying mainly adjusts the proportion of monosaccharides through high-temperature oxidation; roasting introduces components or acidic environments through adjuvants to promote hydrolysis and reactions; steaming triggers degradation and transformation through the effect of moist heat. More importantly, the intensity of processing (temperature, time, and the pH of the excipients) is a key factor influencing the degree of change in the proportion of monosaccharides. This rule provides an important basis for optimizing the processing technology and controlling the quality of polysaccharides in TCM (Wang Z. et al., 2024).

### 4.3 The influence of processing on the chemical structure of polysaccharides in TCM

As a crucial step in the processing of TCM, processing can influence the chemical structure of polysaccharides through various means such as high temperature, moist heat and excipients, thereby altering their physicochemical properties and biological activities. The structural changes of polysaccharides mainly occur at the primary structure level, such as the cleavage and reformation of glycosidic bonds, and the alteration of monosaccharide composition, functional group transformation, such as the oxidation of hydroxyl groups to carboxyl groups, and higher-order structure, such as conformational changes (Guo et al., 2021).

During the processing, high temperature and humid heat environment are prone to cause the cleavage of glycosidic bonds, especially having a significant impact on the thermally unstable β-type glycosidic bonds, and even leading to the formation of new bonds or conformational changes. For Instance, after frying MMF, there was a decrease in the content of β-glycosidic bonds and the appearance of new glycosidic bonds (1,4,6-linked-Glcp) (Liu S. et al., 2024). Steaming PR for 2 h resulted in the destruction of the β-fructan structure (Li et al., 2021c), and the detection of a new glycosidic bond β-1,4-Galp along with an increased β-1,4-Manp (Chen Z. et al., 2022). Similarly, steaming *Chlamys nobilis* (CN) also resulted in a shift in monosaccharide conformation from β type to α type (Yang et al., 2025). Attributed to the hydrolysis and redox reaction of glycosidic bonds at elevated temperatures. This process leads to the degradation or recombination of polysaccharides. To systematically analyze the structural changes induced by processing, a variety of modern analytical techniques are employed for comprehensive characterization from macro to micro levels (Table 3). Processing can also lead to the transformation of functional groups in polysaccharides, such as the oxidation of hydroxyl groups to carboxyl groups. This is exemplified by the increase in the molar ratio of uronic acid after MMF was fried, indicating that the hydroxyl groups of Gal and Glc were oxidized to carboxyl groups of galacturonic acid and Glc UA (Liu S. et al., 2022). Furthermore, the reduction in molecular weight of polysaccharide following the steaming process of PR may be attributed to the development of intermolecular hydrogen bonds between carboxyl groups of uronic acid and other hydroxyl groups, facilitating the breakdown of aggregates. The processing of polysaccharides has a significant impact on their higher structure. In a Congo red experiment, it was found that the crude polysaccharide of CH exhibited a triple helix structure (Hou et al., 2023). However, the conformation of the steamed polysaccharide disappeared, indicating that processing can disrupt the high-order spatial conformation of polysaccharides, possibly due to changes in monosaccharide composition inhibiting helix formation (Li et al., 2021c).

**TABLE 3:** Application of characterization technology of polysaccharide-related structure.

Name	Application	Associated with processing	References
HPSEC/GPC	Used to determine the molecular weight distribution (such as weight average molecular weight Mw, number average molecular weight Mn, dispersion coefficient Mw/Mn) and average molecular weight of polysaccharides	HPSEC/GPC can directly reverse the decrease/increase of molecular weight and the change of distribution width of polysaccharides before and after processing	Han et al. (2025)
GC-MS	Used to identify monosaccharide species and quantitatively analyze the proportion of monosaccharides by peak area	GC-MS can determine whether monosaccharide types are increasing/decreasing before and after processing and the proportion changes	Zhang et al. (2018)
HPLC	It is used for monosaccharide composition analysis, oligosaccharide fragment separation and glycosidic bond type preliminary judgment of thermally unstable monosaccharides	Compared with GC-MS, HPLC is more suitable for rapid quantitative analysis of monosaccharide content changes, and helps to verify the influence on monosaccharide composition before and after processing	Qi et al. (2022)
FT-IR	Used to analyze the characteristic functional groups and glycosidic bond types of polysaccharides	During processing, dehydration of polysaccharide may reduce the intensity of -OH peak, oxidation may increase the -COOH peak, and glycosidic bond breakage may lead to the change of C-O-C peak shape, which directly reflects the difference of functional groups before and after processing	Wu (2021)
NMR	Used to analyze the monosaccharide configuration, connection mode, substituent position and other fine chemical structures of polysaccharides	NMR is the standard to analyze the fine structural changes that may be caused by processing, such as glycosidic bond breakage, configuration transformation and substituent shedding	Wang (2023)
CD	The conformational stability is reflected by the peak position and intensity of CD spectrum, which is used to analyze the secondary/advanced stereoconformation and chiral structure of polysaccharides	It is identified that processing may destroy the spiral structure of polysaccharide or turn amorphous structure into ordered conformation	Zhang et al. (2025b)
SEM	It is used to reveal the relationship between microstructure and function of polysaccharides and optimize the process	SEM visually shows the physical morphological changes of polysaccharide particles that may be broken, the surface becomes rough or aggregates are formed by processing through micron-scale resolution	Mao et al. (2025)
AFM	It is used to analyze the nano-scale morphology of polysaccharides and reflect the stretching/folding state of molecular chains	AFM can observe the molecular chain length of polysaccharide after processing. Or that interaction between chain after processing	Hu et al. (2025)
X-ray	The crystallization of polysaccharide was analyzed, and the crystallinity was calculated by peak area	Reaction processing may destroy the crystallization area of polysaccharide or induce the formation of new crystals	Yue (2023)

Polysaccharides, as biological macromolecules containing multiple hydroxyl groups and glycosidic bonds, are highly sensitive to processing. Processing mainly triggers the cleavage and recombination of glycosidic bonds, the oxidation of functional groups, and changes in higher-order conformations through high temperatures and the action of excipients, ultimately affecting the activity and efficacy of polysaccharides. For instance, the structural changes of polysaccharide after processing of *Rehmanniae Radix Praeparata* (RRP) are closely linked to the transformation of its medicinal properties from “clearing heat” to “enriching blood”. This indicates that the structural change of polysaccharide plays a crucial role in achieving “reducing toxicity and increasing efficiency and changing medicinal properties” during the processing of TCM. This knowledge offers a structural foundation for optimizing processing technology and quality control of TCM.

Various modern analytical techniques have played a crucial role in systematically examining the effects of processing on the chemical structure of TCM polysaccharides. This has resulted in the establishment of a multi-dimensional characterization system that encompasses macroscopic physical properties to microscopic structure-activity.

HPSEC/GPC accurately monitors changes in molecular weight distribution during processing (Han et al.), while GC-MS and HPLC quantitatively analyze changes in monosaccharide composition and proportion (Zhang et al., 2018). FT-IR and NMR reveal fractures (Hong et al., 2021), oxidation, or reconstruction of chemical bonds and glycosidic bond configurations, respectively; CD, SEM, AFM, and XRD are utilized to assess the advanced structural modifications in red bean polysaccharide (Zhang et al., 2025c). XRD analysis indicates a decrease in crystallinity post-enzymolysis, with subsequent physical treatments inducing rearrangement and crystal recovery. This reflects a dynamic “destruction-reconstruction” process during processing. These techniques complement each other, providing a comprehensive understanding of structural changes in polysaccharides, which in turn aids in elucidating changes in biological activity and processing mechanisms. The application of the aforementioned related technologies in the research of polysaccharides and the application of polysaccharide research before and after processing are shown in Table 3.

## 5 The influence of different processing methods on the biological activities related to polysaccharides in TCM

The current research indicates that polysaccharides have demonstrated effects in immunomodulation, antioxidation, hypoglycemic, anticancer, and regulation of intestinal flora. These activities were further enhanced after processing. Table 4 outlines the specific effects of processed Chinese medicine polysaccharides on the mentioned activities.

**TABLE 4:** Effects of different processing methods on related biological activities of polysaccharides in TCM.

Represents TCM	Processing method	Polysaccharide	Cell/animal model	Main mechanism	References
Immunoregulation					
DR	Bran frying	BFDRP	Mice were injected with cyclophosphamide (immunosuppressant) to establish an immunosuppressed model	Bran-fried polysaccharide can promote the production of serum hemolysin in mice and play an immune enhancement role	Cheng et al. (2006)
AMR	Bran frying	BFAMR	Cyclophosphamide-induced mice	The TLR4 receptor on the surface of immune cells is recognized by high proportion of Glc, which activates the TLR4/MyD88/NF-κB pathway and promotes the secretion of cytokines, the increase of Ara content in BFAMR can enhance the phagocytic activity of macrophages, stimulate the release of NO and cytokines, and enhance the immune activity	Wang et al. (2023a)
PR	Steaming	SPRP	RAW264.7	After steaming, the molecular weight of PRP increases. The secretion of cytokines such as NO, TNF-α and IL-6 increased significantly, and the immune activity was enhanced	Wu et al. (2022b)
	Nine steaming and nine processing	NSPRP	RAW264.7	Gal residues promote SCFAs production and indirectly regulate immunity; the carboxyl group of galacturonic acid enhances the binding with immune cell surface receptors through charge interaction, activates NF-κB, TLR4/MyD88 and SCFA-GPR signal pathways, promotes cytokine secretion and enhances immune activity	
	Nine steaming and nine processing	NSPRP	For cyclophosphamide-induced mice	The immune organ index, the effect of promoting the secretion of cytokines and immunoglobulin are more significant, and the immune regulation function is enhanced	Luo et al. (2025a)
NRR	Steaming	SSNRRP	RAW264.7	After steaming, the proportion of Glc increases, which enhances the binding ability with immune cell surface receptors, promotes macrophage activation and cytokine secretion, and can also enhance non-specific immune function by activating monocyte-macrophage system; the α -glucosidic bond structure is more easily recognized by glycosidase on the surface of macrophages, which promotes neutral red phagocytosis and cell proliferation and enhances antioxidant activity	Xing et al. (2021)
Antioxidant activity					
PR	Nine steaming and nine processing	NSPRP	*In vitro* simulated digestion experiment	Hydrolysis occurs in the process of processing and digestion, and it is converted into low molecular weight reducing sugar, which is positively correlated with the antioxidant activities of DPPH and ABTS, and the antioxidant activities are improved	Wang et al. (2024a)
		NSPRP	NA	Steaming leads to partial breakage of glycosidic bonds, and the generated low molecular weight fragments cooperate with aggregated polysaccharides. The high proportion of Ara promotes the contact with free radicals by enhancing the hydrophilicity of molecules, and the generated Glc fragments have strong hydrogen donor ability and enhanced antioxidant activity	Li et al. (2021c)
		NSPRP	NA	The processed polysaccharide with larger molecular weight can inhibit the generation of free radicals through more stable molecular structure, more active sites or stronger free radical trapping ability	Zhang (2021)
		NSPRP	HepG2 cells	After processing, the high molecular weight components have longer molecular chains and more exposed hydroxyl and carboxyl groups, which can provide more active sites to combine with free radicals and directly remove ROS; low molecular weight components can penetrate cell membrane more easily, promote the expression of antioxidant enzymes in cells, and enhance the antioxidant effect synergistically	Liu et al. (2025b)
	Steaming	SPRP	Establishment of oxidative damage model in mice by intraperitoneal injection of D- Gal	After processing, the polysaccharide with larger molecular weight can significantly improve the scavenging ability of DPPH and ABTS free radicals through more stable molecular structure or stronger free radical trapping ability, and has a stronger protective effect on oxidative damage mice	Teng (2022)
CH	Steaming Wine processing	SCHP WCHP	NA	The content of uronic acid in SCHP and WCHP increased, which enhanced the electron transfer of polysaccharide, and the content of Glc in WCHP was higher, which had stronger hydrogen supply capacity and enhanced antioxidant activity	Liu et al. (2024a)
	Enzymolysis	ECHP	NA	ECHP has lower molecular weight, smaller steric hindrance, easier to combine with free radicals, higher content of EH-CDPs uronic acid, enhanced hydroxyl radical scavenging ability and improved antioxidant capacity	Hou et al. (2023)
AS	Steaming	SASP	NA	After steaming, the structure of polysaccharide changed, and the active sites were exposed, which made it easier to interact with free radicals and enhanced its antioxidant activity	Peng et al. (2016)
MMF	Stir-fried	STFMMFP	NA	STFMMFP has lower molecular weight, which is beneficial to its combination with free radicals and enhanced antioxidant activity; in the frying process, part of the side chain structure of STFMMFP was destroyed, and the side chain reduced the steric hindrance less, which was easier to react with free radicals and enhanced the antioxidant activity	Liu et al. (2024b)
GRER	Steaming	SGRERP	NA	The antioxidant activity of SGRERP Ⅱ is significantly stronger than that of component SGR ERP Ⅰ, and the antioxidant activity of small molecular polysaccharide may be enhanced because of its more flexible structure, easier combination with free radicals or participation in redox reactions	Zhao et al. (2017)
MMF	Stir-fried	STFMMFP MMFP	By expose that transgenic zebrafish to metronidazole-induced oxidative stress, an oxidative damage model is constructed	The higher Glc UA content of STFMMFP enables it to provide more hydrogen to react with free radicals and enhance its antioxidant activity. In addition, the higher contents of 1,2-linked-Manp and T-linked-Fucp1,3-linked-Fucp in MMFP also enhanced the antioxidant activity	Liu et al. (2024b)
Hypoglycemic activity					
PR	Nine steaming and nine processing	NSPRP	HepG2 human hepatocellular carcinoma cells/BKS-db mice	Because NSPRP contains a higher proportion of 1)-β-frup-(2 (fructopyranose), its hypoglycemic activity is significantly better than that of raw polysaccharide PRP.	Yu et al. (2024)
DE	Steaming Oil processing roasting	SDEP ODEP RDEP	NA	Inhibition of α -amylase and α -glucosidase to achieve hypoglycemic activity	Tang et al. (2022)
Anticancer activity					
LE	Steaming	HPSLEP	HepG2 (hepatocarcinoma cell) and HeLa (cervical cancer cell)/H22 tumor-bearing mice	The increase of low molecular weight polysaccharide can enhance its ability to penetrate tumor cells and bind to target, and the increase of uronic acid content can enhance the interaction between polysaccharide and tumor cell surface receptors, or improve the anti-cancer effect by regulating immune factorsIL-2, TNF-α and angiogenesis factors VEGF.	Li et al. (2018)
Effect on intestinal flora					
DE	Steaming Oil processing Roasting	SDEP ODEP RDEP	NA	High-temperature treatment may make the DEP structure more susceptible to degradation by intestinal bacteria, disrupt the dense structure of polysaccharides and enhance microbial accessibility. Moreover, the α diversity of polysaccharides in the roasted products was lower than that in the raw products, indicating that polysaccharides can selectively enrich *Bacteroides* bacteria, reduce overall diversity and enhance functional stability	Wu et al. (2022a)
Hepatoprotective effect					
ASR	Fried chaicoal Oil processing Wine processing Sand frying	ASRPC OASRP WASRP SFASR	Acute liver injury model induced by CCl_4_	ASRPC can significantly regulate the activities of ALT and AST, and the other components are also improved to varying degrees	Hua et al. (2014)
	Stir-fried	STFASRP	The liver injury model induced by cefotaxime sodium (CS) combined with lipopolysaccharide (LPS) was established in laying hens	It plays a protective role by regulating arachidonic acid metabolism and mTOR signaling pathway	Wu et al. (2022a)
PMR	Nine steaming and nine processing	NSPMR	NA	NSPMR is a high acidic sugar, which can improve the activities of SOD, GSH-Px and reduce the contents of ROS and MDA in liver tissue. NSPMR and PMRP can significantly reduce the increase of ALT, AST, ALP and LDH induced by APAP. All of them downregulated the mRNA expression of IL-6, IL-1β and TNF-α in liver tissue, and inhibited the inflammatory reaction	Wang et al. (2023b)
Anti-inflammatory activity					
AR	Honey processing	HARP	RAW264.7	Smaller molecular weight may be more conducive to the combination of polysaccharide with cell surface receptors, or easier to be absorbed by cells, thus enhancing its ability to inhibit the release of inflammatory factors; The increase of Glc content in HARP enhanced the anti-inflammatory activity; acetyl may interact with inflammatory cells and enhance anti-inflammatory ability	Liao et al. (2018)

### 5.1 Immunoregulation

**FIGURE 1:**
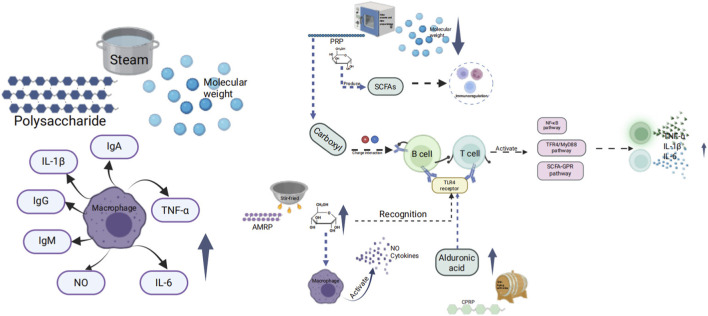
Effects of different processing methods on immune regulation of related polysaccharides.

Immune activity is the ability of substances to influence the immune system, enhancing immune function and balancing immune state. Polysaccharide immune activity primarily involves initiating signal transduction pathways by binding with immune cell surface receptors, which then regulate immune cell proliferation, differentiation, and cytokine secretion, ultimately affecting the body’s immune response (Wang et al., 2023c). The effects of polysaccharides on immune regulation can be observed before and after processing in Figure 1.

Immunocompromised mice show better immune function improvement with the bran-fried DR polysaccharide compared to DR polysaccharide (Cheng et al., 2006). Polysaccharide’s immune activity can be impacted by its molecular weight, with smaller polysaccharide fragments having a greater tendency to bind with immune cells. After undergoing moderate steaming 2–4 times, the molecular weight of PRP significantly increases, leading to the formation of larger particle aggregates, enhance the activation of RAW 264.7 macrophages. When polysaccharides undergo excessive steaming (for more than 6 h), they will undergo depolymerization, resulting in a significant reduction in molecular weight and the dispersion of aggregates. This hinders the effective activation of immune signaling pathways by low-molecular-weight fragments, ultimately leading to a decrease in cytokine secretion and a weakening of immune activity (Wu W. J. et al., 2022). The PRP’s average molecular weight decreased from 7.36 × 10^4^Da to 2.71 × 10^4^Da. Polysaccharides with low molecular weight may have a higher propensity to activate immune signal pathways and improve immune regulation function, attributed to their superior water solubility and bioavailability (Luo W. et al., 2025). In normal macrophages, the low molecular weight fraction (MW <50 kDa) of PRP processed with wine shows a stronger impact on TNF-α secretion than the high molecular weight fraction (MW > 50 kDa) (P < 0.01). It also enhances NO secretion (P < 0.05). However, there is no distinction between the high molecular weight fraction and the low fraction of unprocessed polysaccharide. Consequently, polysaccharide processed with wine displays potent immune activity. Immune activity can be influenced by the monosaccharide composition, as uronic acid, Gal, and Glc have been found to exhibit immune activity. For instance, PRP contains less Gal and galacturonic acid than SPRP and NSPRP. Gal residues can serve as a fermentation substrate for intestinal probiotics, stimulating the production of short-chain fatty acids (SCFAs), and thereby indirectly influencing immune activity; the carboxyl group of Gal UA can increase its binding to the surface receptors of immune cells through electrostatic interactions, thereby activating related signaling pathways, promoting the secretion of cytokines, and enhancing immune activity (Cheng et al., 2006; Wu W. J. et al., 2022). BFAMRP exhibited stronger immune activity compared to AMRP, with higher levels of Glc and Ara but lower levels of galacturonic acid. The elevated Glc content may activate the TLR4/MyD88/NF-κB pathway, resulting in enhanced secretion of cytokines TNF-α, IL-1β, and IL-6 through recognition of the TLR4 receptor on immune cells’ surface. Moreover, Ara rich BFAMRP can boost the phagocytic activity of macrophages and induce the production of NO and cytokines, aligning with BFAMRP’s stronger immunomodulatory impact (Wang D. et al., 2023). The content of Glc in steamed *Notoginseng Radix et Rhizoma* polysaccharide (SNRRP) is higher than NRRP, high proportion of Glc promotes the activation of macrophages and the secretion of cytokines, thereby enhancing immune activity (Xing et al., 2021). After wine processing, the uronic acid content of *Coptidis Rhizoma* polysaccharide (CPRP) increases, which may enhance the interaction of polysaccharides with pattern recognition receptors on T and B lymphocytes, leading to better inhibition of excessive proliferation. Particularly, after wine processing, the suppression of LPS-induced B cell proliferation is more pronounced (Guan et al., 2016).

Different glycosidic bond configurations and spatial structures have varying effects on immune activity. For instance, PRP contains a β-type fructofuranoside bond (FT-IR shows 932cm^-1^ and 809 cm^-1^ characteristic peaks), while the structure is destroied after steaming, potentially relieving immune activity inhibition. Congo red experiment showed that both PRP and SPRP maintain a triple helix conformation, crucial for polysaccharide and immune cell receptor binding (Wu W. J. et al., 2022). This is also confirmed in AMPR and BFAMPR. Both AMPR and BFAMPR have triple helix structure, which is an important basis for the combination of polysaccharides with immune cell receptors and provides structural support for immune activity (Wang D. et al., 2023). Upon analyzing the FT-IR of PRP and NSPRP, it was noted that the crude drug of polysaccharide contained α-glucosidic bonds and β-glucosidic bonds. Subsequent to nine steaming and nine processing, the characteristic peak of α-glucosidic bond vanished, leaving only β-glucosidic bonds. Polysaccharides predominantly consisting of β-glucosidic bonds can be metabolized into active fragments by intestinal flora more easily and are likely to be recognized by immune cells, thereby enhancing immune regulation ability (Wu W. J. et al., 2022). After steaming, NPPR undergoes a structural change where the β-glucosidic bond is broken, and the α-glucosidic bond becomes the main one. This change increases water solubility and bioavailability of the polysaccharide, making it more easily recognized by immune cells. The phagocytic index of RAW264.7 cells after steaming of NRRP polysaccharides (SNRRP) increased from 40.92% to 49.86%, which was significantly higher than NRRP, and this increase is concentration-dependent. The increase in phagocytic index enhances neutral red phagocytosis and cell proliferation. Additionally, a high dosage of SNRRP has the ability to notably increase the carbon clearance index (K), phagocytosis index (α), spleen index, and thymus index in immunosuppressed mice induced by cyclophosphamide (Cy), outperforming NRRP. The elevated Glc content in its structure activates the mononuclear phagocyte system, boosting nonspecific immune function. Structural optimization following steaming could potentially enhance its stability and effectiveness *in vivo* (Xing et al., 2021).

### 5.2 Antioxidant activity

**FIGURE 2:**
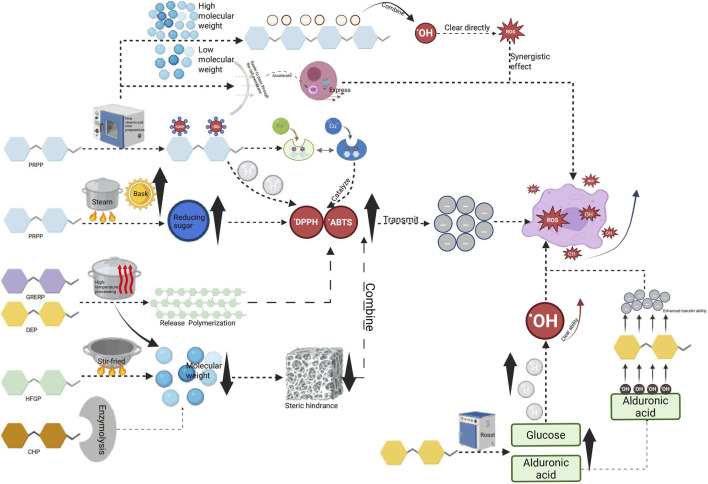
Effects of different processing methods on antioxidant activity of related polysaccharides.

Antioxidant activity is the ability of substances to reduce oxidative damage by inhibiting or delaying oxidation reactions (Wang S. et al., 2022), which can lead to cell damage or diseases like aging, inflammation, and cancer caused by reactive oxygen species (ROS) such as superoxide anion (O^2-^), hydroxyl radical (OH), and hydrogen peroxide (H_2_O_2_) (Mohanta et al., 2022; Zhu et al., 2022). Polysaccharides demonstrate antioxidant activity primarily by boosting the endogenous antioxidant system, inhibiting lipid peroxidation, chelating metal ions, etc., (Bai et al., 2022; Fernandes and Coimbra, 2023). Processing alters the structure of TCM polysaccharides through physical or chemical means, thus impacting their antioxidant activity (Chen et al., 2024). The influence of polysaccharides on antioxidant activity pre- and post-processing is illustrated in Figure 2.

The antioxidant activity of processed TCM polysaccharides is usually more potent than that of unprocessed products, thanks to the combined effects of multiple factors. The antioxidant activity of polysaccharides fluctuates with changes in content pre- and post-processing. For instance, the increase in steaming time leads to the hydrolysis of PRPP during processing and digestion, which is then converted into low-molecular-weight reducing sugars. This leads to a significant increase in the content of reducing sugars, and a higher content of reducing sugars has a positive impact on enhancing antioxidant activity. Therefore, the antioxidant activity of SPRP was found to be stronger than that of PRPP (Wang X. L. et al., 2024). High temperature treatments on *Dioscorea esculenta* (DE) such as steaming and frying resulted in a decrease in polysaccharide content by 22.24% and 44.44% respectively, as some polysaccharides were degraded or dissolved; in contrast, roasting treatment led to an increase in polysaccharide content by 21.33%. This is thought to be because the high temperature promoted the release or repolymerization of polysaccharides, generating more fragments with antioxidant activity and enhancing overall antioxidant capabilities (Liu C. et al., 2024). The study found that unprocessed CH had the highest polysaccharide content but the lowest antioxidant activity. The structure of its polysaccharides might be limited in antioxidant capacity because it has not undergone thermal activation, and the active sites are either wrapped or not exposed, but antioxidant activities (ORAC, DPPH, FRAP) significantly increased. This indicates that moderate thermal processing can alter the polysaccharide structure, making its active site more accessible to interact with free radicals and improve antioxidant activity (Peng et al., 2016). The change in antioxidant activity is closely linked to the change in molecular weight. For example, the average molecular weight of stir-fried *Hordei Fructus Germinatus* polysaccharide (STFHFGP) is 8652Da, which is lower than that of *Hordei Fructus Germinatus* polysaccharide (HFGP) at 9534Da. The study revealed that the higher molecular weight of HFGP hinders its interaction with free radicals, thus reducing its antioxidant effectiveness. On the other hand, the lower molecular weight of STFHFGP allows for better binding with free radicals, leading to enhanced antioxidant activity (Liu S. et al., 2022). Low molecular weight polysaccharides in CH exhibited stronger antioxidant activity compared to high molecular weight polysaccharides. Enzymatically treated CHP (ECHP) had the lowest molecular weight (1,531.50 kDa) and showed the highest hydroxyl radical scavenging ability (IC = 0.25 mg/mL), significantly outperforming other samples. In contrast, the molecular weight of wine-steamed CHP (WCHP) is the highest, reaching 2,526.00 kDa. Its DPPH and ABTS free radical scavenging abilities are relatively weak, and its antioxidant activity is also relatively low (Hou et al., 2023). The study on PR and NSPR revealed that the molecular weight of unsteamed polysaccharide was 6.06 kDa. The molecular weight remained stable for the first four steaming sessions, but significantly increased from the fifth steaming, reaching 75.26 kDa. The polydispersity index Mw/Mn also increased, indicating aggregation of polysaccharides. The antioxidant activity (DPPH, ABTS, hydroxyl radical scavenging ability) gradually increased with the increase in the number of steaming cycles. Especially after the fourth steaming, the antioxidant capacity significantly improved (Li et al., 2021c). The molecular weight of PR before and after steaming is 4.35 × 10^3^Da, indicating a small molecular polysaccharide. On the other hand, the molecular weight of SPRP is 4.24 × 10^4^ Da, which belongs to relatively large molecular polysaccharides. The larger molecular weight of polysaccharides results in stronger antioxidant activity (Teng, 2022) Zhang, (2021) research also confirmed this view. Moreover, this study also demonstrated that the increase in the molecular weight of polysaccharides during the preparation process might be attributed to the intermolecular aggregation caused by the Maillard reaction (Zhang, 2021). Zhao and others used sephadexG-75 gel chromatography column to classify the neutral polysaccharides present in dried ginseng and red ginseng processed at different temperatures based on their molecular weights (Zhao et al., 2017). The antioxidant activity of NSPRP and nine preparation is closely related to their molecular weight. NSPRP is composed of two main components with weight average molecular weights of 196857Da (component 1) and 13653Da (component 2) respectively. The overall molecular weight is significantly increased compared to PRP due to intermolecular aggregation during processing. The high-molecular-weight component (196,857 Da) has a longer molecular chain, can bind to more oxidation sites, and has enhanced antioxidant activity (Liu Y. B. et al., 2025).

The change in monosaccharide composition or content before and after processing affects the antioxidant activity. For example, CFP processed by different methods showed that CFP has the highest proportion of Glc (89.82%), but the weakest antioxidant activity (DPPH scavenging rate of 54.27%, 1.8 mg/mL). It is speculated that the high proportion of Glc may create a dense structure that hinders the exposure of active sites. HCFP, which is hot air dried CFP, has relatively high contents of Ara (10.43%) and Gal (5.59%), resulting in the strongest DPPH and ABTS scavenging abilities (IC_50_’s of 0.6 mg/mL and 0.72 mg/mL respectively). These two monosaccharides may enhance electron transfer ability through hydroxyl groups or form a looser glycosidic bond structure. ECFP, which is enzymatic hydrolysis CFP, has the highest content of galacturonic acid (16.53%), leading to outstanding hydroxyl radical scavenging ability, possibly due to the reactivity of the carboxyl group of uronic acid with free radicals (Ma et al., 2024). Four polysaccharides from unprocessed DEP and those processed by steaming, frying, and roasting were compared for their structure and antioxidant activity. All polysaccharides were found to be composed of fructose, Glc, Man, and Gal, but with varying molar ratios. The roasted polysaccharide showed a significant increase in Glc content, which could enhance hydrogen supply ability and improve hydroxyl radical scavenging ability. Steamed and roasted polysaccharides had significantly higher uronic acid content compared to unprocessed and oil-fried samples. The carboxyl group of uronic acid can enhance the electron transfer ability of polysaccharides, which is positively correlated with antioxidant activity. This may explain the higher antioxidant activity observed in the roasting group (Liu C. et al., 2024). The proportion of Man decreased gradually with the increase of steaming times in PRP and NSPRP (NSPRP-0 was 51.2%, NSPRP-9 was 17.5%). The proportion of Glc increased from 21.4% to 46.5%, and the proportion of Ara increased from 0.46% to 5.89%. The proportion of Gal decreased first and then increased, stabilizing after the fourth steaming. Correlation analysis showed that the scavenging capacity of ABTS and hydroxyl radicals was negatively correlated with Man content, and positively correlated with Ara content. The increase in the ratio of Ara to Glc enhances the antioxidant activity (Li et al., 2021c), and Wu and Liu’s research also confirmed this point (Liu Y. B. et al., 2025; Wu et al., 2021).

The monosaccharide composition of the acidic polysaccharide isolated from fried MMF(FMMFP) includes fucose, Rha, Gal, Man, Gal UA, and mannuronic acid. The higher content of Glc UA in MMFP polysaccharides (MMFAP) contributes positively to their antioxidant activity due to the specific glycosidic bond type (Liu S. et al., 2024).

Before processing, PRP was mainly composed of fructose (93.57%) and a small amount of Glc (6.43%). After processing, the composition became more complex with the addition of Rha (0.50%), Gal (0.05%), Man (0.83%), and Gal UA (0.83%). The presence of Gal UA and Glc UA in the processed polysaccharide can inhibit the generation of free radicals by chelating iron ions. The synergistic effect of these various monosaccharides may enhance the binding efficiency with free radicals, thereby increasing its antioxidant activity (Zhang, 2021). The glycosidic bond and spatial structure of polysaccharides undergo different changes depending on the processing method used, which in turn affects the antioxidant activity of the polysaccharide. For example, nine steaming and nine processing results in a rough surface with wrinkles, cracks, and pores appearing on the PRP, while seven steaming and seven processing leads to a significant destruction of the structure. This structural change increases the contact area of the polysaccharide with digestive juice and enzymes, allowing more antioxidant components to participate in reactions and enhancing the overall antioxidant activity (Wang X. L. et al., 2024). The ratio of side chain (1,2- linked mannose residue) in MMFP exceeds that of FMMFP, and the frying process might disrupt certain side chain structures. FMMFP possessing fewer side chains, enhanced antioxidant capacity (Liu S. et al., 2022).

Processed CH using different techniques showed that CHP and enzymatic CHP(ECHP) had a triple helix conformation, with ECHP showing stronger activity compared to CHP. However, hot air drying CHP(HACHP), wine steamed CHP(WCHP), and high pressure steaming CHP(HPSCHP) did not have a triple helix structure, yet exhibited higher antioxidant activity. It is believed that the rigid triple helix structure of CHP may limit its interaction with free radicals, while a random curly structure is more conducive to reactions. Microstructure analysis revealed that WCHP had the densest microstructure and lowest activity, while HACHP had a loose structure and the highest activity. The loose structure of HACHP can increase specific surface area and enhance contact efficiency with free radicals (Hou et al., 2023).

The study examined the functional groups of polysaccharides from DR (DRP) heated at different temperatures. It was discovered that the antioxidant activity was attributed to carbonyl, amino, and heterocyclic structures formed through the Maillard reaction. FT-IR analysis revealed O-H stretching vibration peaks at 3,700–3,000 cm^-1^ and C=O or C=N peaks at 1,650–1,600 cm^-1^ in all DR polysaccharides. The low-temperature products retained more hydroxyl groups without over-oxidation, leading to increased antioxidant activity through hydrogen atom transfer mechanism for free radical removal (Li et al., 2021b). The study on the antioxidant activity of DE through heating processing revealed that the polysaccharides maintained their basic functional groups, such as the hydroxyl group and glycosidic bond, indicating that their antioxidant ability was not compromised. The presence of the characteristic peak at 800–950 cm^-1^ confirmed that all polysaccharides retained their stable pyranose structure after thermal processing, ensuring they still possessed antioxidant activity (Liu C. et al., 2024). High temperature steam treatment of *Adenophora stricta* (AS) can break glycosidic bonds in polysaccharides, leading to the formation of low molecular weight fragments. These fragments have enhanced antioxidant activity as they can easily interact with free radicals. Additionally, steam treatment can disrupt the tight aggregation of polysaccharides, exposing more antioxidant active groups such as hydroxyl. Hydroxyl can directly scavenge free radicals, resulting in increased antioxidant activity (Peng et al., 2016). Unprocessed PRP has a flexible chain conformation with an α value of 0.59. After the fifth steaming, the α value decreased to 0.40–0.49, indicating a change to a spherical shape due to molecular chain folding from aggregation. The microscopic morphological difference shows that PRP-0 is a loose sheetlike structure that becomes tighter with increased steaming. The spherical conformation and aggregated structure may stabilize intermediates during radical scavenging, while the tight structure enhances the exposure efficiency of antioxidant groups like hydroxyl groups to improve antioxidant capacity (Li et al., 2021c).

### 5.3 Hypoglycemic activity

Hypoglycemic activity is the ability of natural products, chemicals, and other substances to lower high blood sugar levels and maintain blood sugar balance by regulating glucose metabolism in the body. This involves interfering with the absorption, utilization, synthesis, or breakdown of sugar to bring blood sugar levels back to normal (Gong et al., 2022; Ji et al., 2023).

**FIGURE 3:**
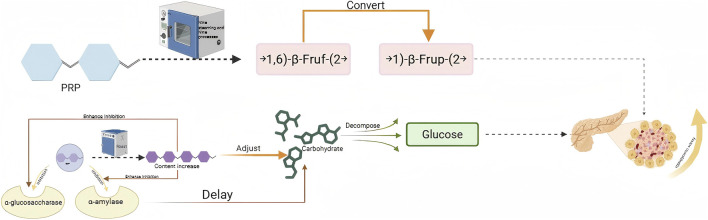
Effects of different processing methods on hypoglycemic activity of related polysaccharides.

Polysaccharides from TCM primarily exhibit hypoglycemic activity by inhibiting carbohydrate digestion and absorption, regulating enzyme activities involved in glucose metabolism, and modulating intestinal flora pathways. The effects of related polysaccharides on hypoglycemic activity before and after processing are shown in Figure 3.

The physicochemical properties and structures of polysaccharides, were found to change after processing, indirectly affecting their hypoglycemic activity. In the study of PRP and NSPRP, it was discovered that the specific glycosidic bond type and monosaccharide configuration transformation were key determinants of hypoglycemic activity. NSPRP, compared with PRP, NSPRP exhibited significantly better hypoglycemic activity (Yu et al., 2024).

The hypoglycemic activity of steamed, roasted, oil fried, and unprocessed DEP was compared, and it was found that the activity was mainly due to the inhibition of α-amylase and α-glucosidase (Tang et al., 2022), Both enzymes help delay the breakdown of carbohydrates into glucose, thereby reducing blood sugar levels. The total sugar content was positively correlated with activity, with roasted polysaccharide having the highest total sugar content and the strongest inhibitory activity on α-amylase and α-glucosidase (Mukherjee et al., 2022); despite this, the oil fried group exhibits the lowest total sugar content and the weakest hypoglycemic activity, indicating that the preservation of total sugar in polysaccharide might be the determining factor in affecting hypoglycemic activity. A higher total sugar content could potentially provide more action sites for enzyme inhibition (Liu C. et al., 2024).

### 5.4 Anticancer activity

**FIGURE 4:**
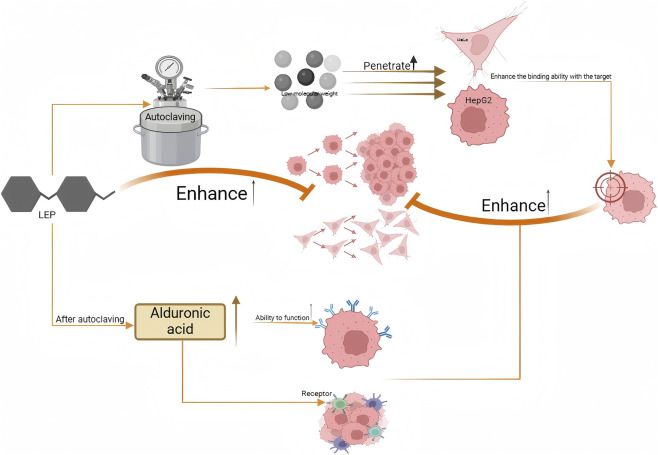
Way of exerting anticancer effect of high-pressure steam LEP.

Cancer is a malignant disease characterized by abnormal cell proliferation, caused by genetic mutations, environmental carcinogens, and chronic inflammation (Wang et al., 2024). The activation of proto-oncogenes and inactivation of tumor suppressor genes lead to the formation of malignant tumors with unlimited growth, invasion, and metastasis (Li N. et al., 2021; Shang et al., 2024). The anticancer activity of polysaccharides is achieved through various mechanisms such as immunomodulation, direct inhibition of tumor cells, and regulation of signal pathways. For example, *in vitro* experiments with *Lentinula Edodes* (LE) polysaccharides (LEP) showed that high-pressure steaming LE polysaccharide had a significantly higher inhibitory rate on liver cancer cells (HepG2) and cervical cancer cells (HeLa) compared to regular LE polysaccharide (Li et al., 2018). Studies have shown that the molecular weight of polysaccharides can affect their anti-cancer efficacy. Polysaccharides with lower molecular weights exhibit better anti-cancer activity than those with higher molecular weights. The uronic acid content of HPSLEP was 30.87%, significantly higher than LEP’s 19.07%. The neutral sugar content decreased from 84.35% to 64.63%, and the protein content decreased from 10.00% to 7.04%. Glc UA is a key regulator of water solubility and biological activity of polysaccharides. An increase in its content may be related to the interaction with tumor cells, or it may enhance the anti-cancer effect by regulating related factors such as immunity. The higher sugar aldehyde content in the high-pressure steamed LEP is consistent with its stronger immune regulation and tumor suppression activity. The higher uronic acid content in HPSLEP aligns with its stronger immunomodulatory and tumor inhibitory activities. The results of further research revealed that both LEP and HPSLEP exhibit typical absorption peaks of polysaccharides and pyranose ring structures, indicating that the basic skeleton of the pyranose ring of LEP remains intact after high pressure treatment. The study suggests that the anticancer activity of LEP is dependent on the pyran ring skeleton connected by its glycosidic bond, which is preserved in HPSLEP. Consequently, HPSLEP retains its anticancer activity, with additional enhancement from changes in molecular weight and uronic acid content. The effects of LEP on anticancer activity before and after processing are shown in Figure 4.

### 5.5 Hepatoprotective effect

**FIGURE 5:**
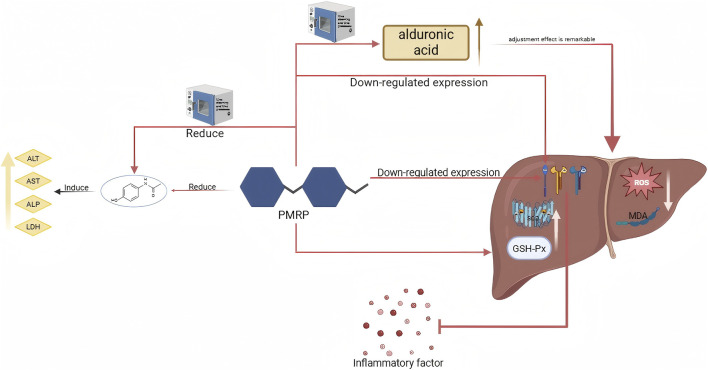
Protective effect of PRP prepared by nine steaming and nine processing on liver.

Take ASR as an example (Hua et al., 2014), This article mainly describes the protective effect and potential mechanism of fried charcoal ASRP(FASRP), wine processing ASRP(WASRP), oil frying ASRP(OASRP) and soil frying ASRP(SLFASRP) on CCl_4_ induced liver injury, the results show that, the activities of ALT and AST in liver injury group were significantly increased, but the activities of these two enzymes were significantly decreased in each polysaccharide treatment group, especially in ASRPC, which was close to the normal level (Sieminska-Kuczer et al., 2022). Hepatocyte swelling, necrosis, and inflammatory infiltration were observed in the liver injury. The ASRPC group showed liver tissue morphology closest to the control group, indicating the best damage repair effect. SLFASRP, WASRP, and OASRP also demonstrated varying degrees of improvement. All processed ASRPs were able to reverse the metabolic disorder caused by liver injury, with ASRPC showing the most significant regulatory effect on most markers, restoring abnormal metabolite levels to near-normal states. In a study on PMR polysaccharide (PMRP) and nine steaming and nine processing PMRP(NSPMRP), it was found that (Wang Y. et al., 2023) PMRP and NSPMRP can enhance the activities of SOD and GSH-Px, while reducing the levels of ROS and MDA in liver tissue. RPMPs exhibit a slightly stronger antioxidant effect, possibly due to their high acid sugar content. High doses of PMRP and NSPMRP significantly decrease the levels of ALT, AST, ALP, and LDH caused by APAP, with similar effects between the two groups. Furthermore, both formulations downregulate the mRNA expressions of IL-6, IL-1β, and TNF-α in liver tissue, thereby suppressing the inflammatory response. The effects of related PMRP on hepatoprotective effect before and after nine steaming and nine processing are shown in Figure 5 H&E staining demonstrated that the high dose group of stir-fried ASRP (STFASRP) could ameliorate hepatocyte necrosis, vacuolation, and central vein dilatation, resembling normal liver tissue. The liver protection mechanism of STFASRP differs from ASRP (Wu F. L. et al., 2022), as it regulates arachidonic acid metabolism and the mTOR signaling pathway involving nine different metabolites.

### 5.6 Anti-inflammatory and other activities

**FIGURE 6:**
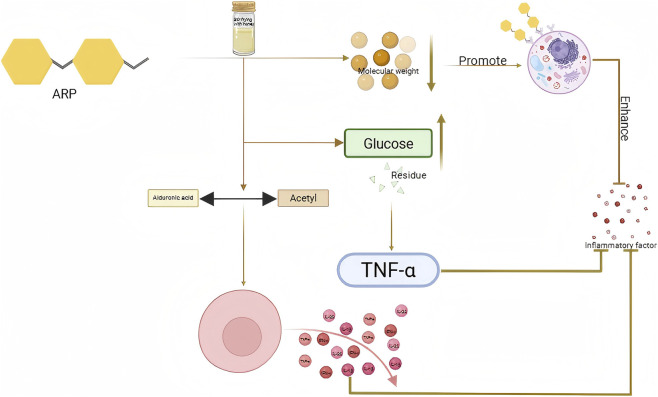
Ways to exert anti-inflammatory activity of honey-processed ARP.

This paper compares the structural differences and anti-inflammatory activities *in vitro* between honey-processed AR polysaccharides (HARP) and AR polysaccharides (ARP) (Liao et al., 2018; Zhu et al., 2025), the study focuses on molecular weight, monosaccharide composition, and functional groups. Results show that HARP has a smaller molecular weight than ARP and exhibits better anti-inflammatory activity. Both HARP and ARP are composed of Man, Glc, Xyl, Ara, Glc UA, and Rha in terms of monosaccharide composition, the proportion of glucose in HARP is 28.34%, which is higher than 12.83% in ARP. This is because during the honey-roasting process, Glc combines with polysaccharides, and other monosaccharides undergo degradation at high temperatures. The structural characteristics of the high Glc content in HARP may enhance its anti-inflammatory activity by participating in inflammatory signaling pathways. FT-IR (Infrared signal at 1,625.77 cm^-1^) and ^1^H-NMR (The NMR signal at δ2.0 ppm) analysis showed higher levels of uronic acid and acetyl group in HARP compared to ARP, which can enhance the anti-inflammatory effect by regulating cytokine secretion and affecting the interaction between polysaccharides and inflammatory cells (Yuan et al., 2023). The effects of ARP on anti-inflammatory activity before and after processing are shown in Figure 6.

The physicochemical properties of PRP were altered through steaming, resulting in enhanced antioxidant activity and improved memory disorder by regulating oxidative stress, inflammatory reaction, and synaptic function (Bian et al., 2022). Through a process of two steaming and two sun-drying, polysaccharides in PRP were found to improve their anti-aging activity by breaking glycosidic bond, adjusting polymerization degree, or synergizing with reducing sugar components (Li et al., 2023).

### 5.7 Effect on intestinal flora

Intestinal flora consists of various microbial communities, such as bacteria, fungi, and viruses, that reside in the human intestines (Li et al., 2025), these microorganisms have a symbiotic relationship with their hosts and play important roles in digestion, absorption, immune regulation, and metabolic balance. Polysaccharides can influence the composition and function of intestinal flora through their structural characteristics. In turn, intestinal flora can metabolize polysaccharides to produce active products that impact the host’s health (Cheng et al., 2022; Xue H. et al., 2025). This bidirectional interaction is crucial for immune regulation, metabolic balance, and disease prevention (Zhang et al., 2022).

**FIGURE 7:**
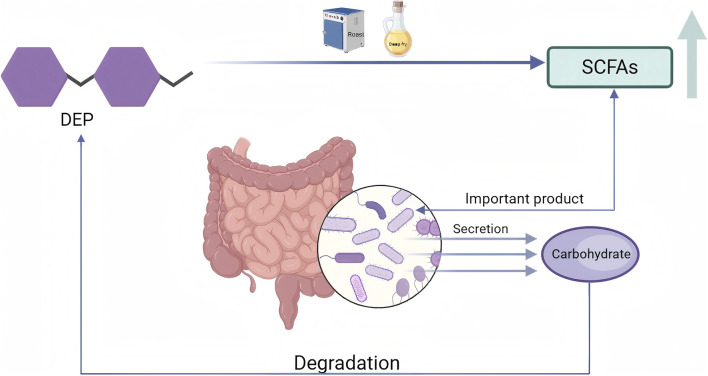
Effect of roasted and oil processed DEP on intestinal flora.

The study found that DEP undergo changes during *in vitro* digestion and fecal fermentation, showing a close relationship between polysaccharide structure and intestinal flora regulation (Liu C. et al., 2025), the reducing sugar content of all unprocessed and heat-treated DEP increased significantly during digestion, indicating partial degradation of the molecular structure. In the fecal fermentation stage, the reducing sugar and total carbohydrate contents further decreased significantly, suggesting that intestinal flora can continuously degrade polysaccharides using carbohydrate active enzymes as carbon sources. The SCFAs content in fermented fried and roasted DEP was significantly higher than in the unprocessed group (P < 0.05). SCFAs, mainly acetic acid, propionic acid, and n-butyric acid, are important metabolites of intestinal flora. N-butyric acid, the main energy source for colon cells, can protect the intestinal mucosa and regulate immunity. Propionic acid can regulate liver metabolism, and acetic acid is involved in the energy metabolism of peripheral tissues. The SCFAs content in polysaccharides varied depending on the heat treatment, with the highest levels found in the oil-fried group. This reflects how structural differences influence bacterial metabolic pathways, as polysaccharides with looser structures are more easily broken down into SCFAs by flora (Zhou et al., 2022). The Shannon index was lowest in the fried group, possibly due to the simpler structure caused by a higher proportion of low molecular weight polysaccharides being utilized by only a few dominant bacteria. The structure of polysaccharides, including monosaccharide composition and molecular weight, can influence the production of short-chain fatty acids (SCFAs) during degradation, which in turn impacts the interaction between gut flora (Yang W. et al., 2022). For instance, acetic acid produced by *Bacteroides* from polysaccharide degradation can stimulate the growth of Bifidobacterium, creating a synergistic metabolic network. In contrast, polysaccharides in the roasted group may be more easily broken down into butyric acid by *Clostridium* due to high temperatures, enhancing the anti-inflammatory effects (Wang T. et al., 2022). The effects of DEP on effect on intestinal flora before and after processing are shown in Figure 7.

## 6 Conclusion

Processing plays a crucial role in the processing of TCM, affecting the content, structure, and biological activity of polysaccharides through various factors such as heat, auxiliary materials, and microbial metabolism. Its mechanism of action may be complex, but there are common patterns. This paper provides a systematic review of the regulatory effect of processing on polysaccharides, with the following main conclusions.

The influence of processing on the polysaccharide content shows bidirectional regulation and process dependence. Stir-frying can both promote cell wall destruction and improve dissolution through high temperature, but it can also lead to degradation and carbonization. Roasting introduces foreign ingredients or creates a reaction environment with the help of auxiliary materials, such as honey, wine and vinegar, potentially increasing or decreasing the polysaccharide content. Steaming, under moderate conditions, can promote the accumulation of polysaccharides, long-term treatment will lead to Maillard reaction and hydrolysis, resulting in a decrease in content. On the other hand, fermentation, microwave, and other methods can cause dynamic changes in content through biotransformation or thermal effects. Overall, processing affects the polysaccharide content of medicinal materials through multiple pathways such as altering their physical texture, destroying cell structures, and triggering chemical reactions. The effect is highly dependent on process parameters (temperature, time, and excipients) as well as the inherent characteristics of the medicinal materials themselves.

The modification of polysaccharide structure through processing is the basis of its functional improvement, which is mainly reflected in three aspects. The molecular weight of polysaccharides from processed TCM generally decreases due to the degradation of glycosidic bonds under the influence of heat, acid and moisture. The proportion of monosaccharide composition and functional groups changes mainly. The stir-frying method may adjust the ratio of uronic acid to neutral sugar, the honey processing method may introduce exogenous monosaccharides or promote hydrolysis, and steaming may cause the breakage of sugar chains, and the increase of functional groups such as carboxyl and acetyl groups further enhances their reactivity. The higher-level structures, including primary glycosidic bond connections, secondary triple helix conformations, and higher-level aggregation states, change, directly affecting the recognition and binding with biological receptors.

Processing achieves the directional enhancement of the biological activity of polysaccharides through structural modification. The immunomodulatory activity was improved by appropriately reducing the molecular weight, increasing the proportion of specific monosaccharides, and retaining the triple helix structure; the antioxidant activity benefits from the optimization of molecular weight, the increase of reducing monosaccharide ratio, the destruction of rigid structure and the introduction of new functional groups; the hypoglycemic activity is closely related to the specific glycosidic bond configuration, formation and total sugar content; anti-cancer activity can achieve synergy by reducing molecular weight, increasing uronic acid ratio and retaining pyran ring skeleton; hepatoprotective effect was significantly enhanced by regulating arachidonic acid metabolism and mTOR signaling pathways; anti-inflammatory and intestinal flora regulation activities mediate immunomodulatory effects by reducing molecular weight, optimizing monosaccharide composition and increasing functional groups, and by using microbial metabolites SCFAs, especially butyric acid.

In summary, the impact of processing on polysaccharides involves a continuous process of macro content change, microstructure remodeling, and enhancement of biological function. The core mechanism includes physical, chemical, and biological transformation to regulate the molecular characteristics of polysaccharides, optimize their interaction with biological systems, and enhance pharmacological activity.

## 7 Perspective

Despite the shift from basic content determination to structure-activity correlation in current research, there remains ample opportunity for further exploration in this field. To advance processing research towards precision, systematicity, and intelligence, future efforts should concentrate on the following directions (Chen et al., 2023).

We need to enhance real-time monitoring and precise control of polysaccharide structure changes during processing. Current research mostly focuses on analyzing final products, but in the future, we should develop a strategy that combines online spectroscopy technology with process analysis technology to track the dynamic changes of key parameters like molecular weight and functional groups of polysaccharides in real time. This will help in creating a “time-temperature-structure” correlation map and enable dynamic optimization and feedback control of process parameters using artificial intelligence algorithms, moving away from traditional empirical quality control methods.

Comprehensive analysis of the structure-activity relationship between specific structural fragments formed during processing and their corresponding biological activities is essential. By utilizing preparative chromatography and activity-oriented separation technology, active oligosaccharide fragments or homogeneous polysaccharide components can be accurately isolated post-processing. Advanced technologies like molecular docking and surface plasmon resonance can then be employed to determine the interaction mode with specific targets, providing insight into which structures drive which activities at a molecular level.

Systematically uncover the mechanism of polysaccharide action through the intestinal flora-immune axis. Utilize metagenomics, metatranscriptomics, and metabonomics methods to identify the metabolic pathways and characteristic products of different processed polysaccharides by specific strains accurately. Investigate how microbial metabolites, such as butyric acid, regulate intestinal immunity and mediate distal effects through the intestinal-liver axis and intestinal-brain axis. Develop a comprehensive pathway network of “processed polysaccharide-structural optimization-microbial metabolism-immune regulation.

It is essential to develop a comprehensive intelligent evaluation system that encompasses processing technology, structure, activity, and efficacy. This system should incorporate process parameters, various levels of structural information, *in vitro* and *in vivo* data, and establish an optimization model that utilizes artificial intelligence and systematic pharmacology methods for predictive purposes. This will enable the precise design of processing technology based on target pharmacodynamics, leading to the advancement and standardization of Chinese medicine processing.

By delving deep into this direction, we aim to uncover the scientific essence of processing and regulating polysaccharides, and ultimately achieve precise process design informed by clinical effectiveness. This will drive innovative development and application of Chinese medicine processing research.

## Glossary

TCM: traditional chinese medicine
Glc: glucose
Xyl: xylose
Ara: arabinose
Fuc: fucose
Rha: rhamnose
Gal: galactose
Man: mannose
Gal UA: Galactouronic acid
Glc UA: Glucuronic acid
AMR: *Atractylodis Macrocephalae Rhizoma*
AMRP: *Atractylodis Macrocephalae Rhizoma* polysaccharide
BFAMRP: Bran frying *Atractylodis Macrocephalae Rhizoma* polysaccharide
CF: *Corni Fructus*
CFP: *Corni Fructus* polysaccharide
HCFP: Hot air dried *Corni Fructus* polysaccharide
ECFP: Enzymatic *Corni Fructus* polysaccharide
TP: *Typhae Pollen*
STF: Stir-fried
SLF: Soil frying
SF: Sand frying
BF: Bran frying
RF: Rice frying
HP: Honey processing
W: Wine processing
SP: Salt processing
V: Vinegar processing
S: Steaming
NS: Nine steaming and nine processing
F: Fermentation
AMRC: *Atractylodis Macrocephalae Rhizoma* charcoal
CFC: *Corni Fructus* charcoal
TPC: *Typhae Pollen* charcoal
RRR: *Rhei Radix et Rhizoma*
RRRC: *Rhei Radix et Rhizoma* charcoal
ABR: *Achyranthis Bidentatae Radix*
ABRC: *Achyranthis Bidentatae Radix* charcoal
ASR: *Angelicae Sinensis Radix*
ASRC: *Angelicae Sinensis Radix* charcoal
ASRP: *Angelicae Sinensis Radix* polysaccharide
WASRP: Wine processing *Angelicae Sinensis Radix* polysaccharide
STFASRP: Stir fried *Angelicae Sinensis Radix* polysaccharide
OASRP: Oil processing *Angelicae Sinensis Radix* polysaccharide
GF: *Gardeniae Fructus*
GFC: *Gardeniae Fructus* charcoal
AR: *Astragali Radix*
ARP: *Astragali Radix* polysaccharide
CR: *Codonopsis Radix*
OR: *Ophiopogonis Radix*
PRA: *Paeoniae Radix Alba*
DR: *Dioscoreae Rhizoma*
DRP: *Dioscoreae Rhizoma* polysaccharide
GGEC: *Galli Gigerii Endothelium Corneum*
CBR: *Cibotii Rhizoma*
SPR: *Scrophulariae Radix*
NRR: *Notoginseng Radix et Rhizoma*
NRRP: *Notoginseng Radix et Rhizoma* polysaccharide
SNRRP: Steaming *Notoginseng Radix et Rhizoma* polysaccharide
AHR: *Anemarrhenae Rhizoma*
BR: *Bupleuri Radix*
CS: *Cuscutae Semen*
EF: *Epimedii Folium*
UL: *Urticadioica* L
LB: *Lilii Bulbus*
POR: *Polygonati Odorati Rhizoma*
EBF: *Eriobotryae Folium*
MOR: *Morindae Officinalis Radix*
SR: *Scutellariae Radix*
PR: *Polygonati Rhizoma*
PRP: *Polygonati Rhizoma* polysaccharide
SPRP: Steaming *Polygonati Rhizoma* polysaccharide
NSPRP: Nine steaming and nine processing *Polygonati Rhizoma* polysaccharide
LLF: *Ligustri Lucidi Fructus*
SCF: *Schisandrae Chinensis Fructus*
PMR: *Polygoni Multiflori Radix*
PMRP: *Polygoni Multiflori Radix* polysaccharide
NSPMRP: Nine steaming and nine processing *Polygoni Multiflori Radix* polysaccharide
CPR: *Coptidis Rhizoma*
CPRP: *Coptidis Rhizoma* polysaccharide
DE: *Dioscorea Esculenta*
DEP: *Dioscorea Esculenta* polysaccharide
ODEP: Oil processing *Dioscorea Esculenta* polysaccharide
RDEP: Roasting *Dioscorea Esculenta* polysaccharide
SDEP: Steaming *Dioscorea Esculenta* polysaccharide
HFG: *Hordei Fructus Germinatus*
HFGP: *Hordei Fructus Germinatus* polysaccharide
STFHFGP: Stir-fried *Hordei Fructus Germinatus* polysaccharide
RRP: *Rehmanniae Radix Praeparata*
CN: *Chlamys nobilis*
CH: *Cistanches Herba*
CHP: *Cistanches Herba* polysaccharide
ECHP: Enzymatic *Cistanches Herba* polysaccharide
OCHP: Oil processing *Cistanches Herba* polysaccharide
WCHP: Wine processing *Cistanches Herba* polysaccharide
HCHP: Hot air dried *Cistanches Herba* polysaccharide
HPSCHP: High pressure steaming *Cistanches Herba* polysaccharide
CBF: *Chebulae Fructus*
RRER: *Rubiae Radix et Rhizoma*
SPF: *Sophorae Fructus*
EDF: *Euodiae Fructus*
GRER: *Glycyrrhizae Radix Et Rhizoma*
PAR: *Pseudostellariae Radix*
ASB: *Allii Sativi Bulbus*
GRR: *Ginseng Radix et Rhizoma*
MYS: *Myristicae Semen*
CSF: *Citri Sarcodactylis Fructus*
GRER: *Ginseng Radix et Rhizoma Rubra*
GRERP: *Ginseng Radix et Rhizoma Rubra* polysaccharide
SGRERP: Steaming *Ginseng Radix et Rhizoma Rubra* polysaccharide
MMF: *Massa Medicata Fermentata* polysaccharide
MMP: *Massa Medicata Fermentata* polysaccharide
FMMP: Fried *Massa Medicata Fermentata* polysaccharide
AS: *Adenophora stricta* Miq
ASP: *Adenophora stricta* Miq polysaccharide
SASP: Steaming *Adenophora stricta* Miq polysaccharide
LE: *Lentinula Edodes*
LEP: *Lentinula Edodes* polysaccharide
HPSLEP: High pressure steaming *Lentinula Edodes* polysaccharide
ALT: Alanine Aminotransferase
AST: Aspartate Aminotransferase
LDH: Lactate Dehydrogenase
APAP: Acetaminophen
SCFAS: Short-Chain Fatty Acids
